# Safety education at research facilities with radiation sources and short-term facility users – Current design and practice and possibilities for improvement

**DOI:** 10.1016/j.heliyon.2024.e32675

**Published:** 2024-06-07

**Authors:** Åsa Ek, Kerstin Eriksson, Jonas Borell

**Affiliations:** a Ergonomics and Aerosol Technology, Department of Design Sciences, LTH, Faculty of Engineering at Lund University, PO Box 118, SE-221 00, Lund, Sweden; b RISE Research Institutes of Sweden, Division of Safety and Transport, Department of Safety Research, Scheelevägen 17, SE-223 70, Lund, Sweden

**Keywords:** Human factors, System safety, Learning, Safety training, Socio-technical system, Competence requirements

## Abstract

Research facilities such as spallation sources and synchrotrons generate radiation for use in atomic-level or molecular-scale experiments. These facilities can be viewed as complex safety-critical systems. An important aspect of the safety management of such systems is the short safety education and training programme the users are required to undergo in order to gain facility access. As research on the topic is limited, this study aimed to increase the knowledge about current education design and practice using the perspectives of safety science and pedagogy. Study objectives were to identify preconditions that impact the safety education design, to describe current design and practice of the safety education, and to identify weaknesses and possibilities for improvement. Site visits with a total of 20 interviews were performed at three research facilities. The results show the need for sufficient resources to maintain learning activities for users, provide pedagogical continuing education for educators, and maintain safety culture-enhancing activities to meet the challenges of having large numbers of short-term facility users. Increased focus should be placed on safety-related competence needs and the mapping of these to match the competence of individual users. New thinking and innovation can benefit the design and provision of such education activities, based on both socio-technical system and system safety perspectives.

## 1 Introduction

Research facilities and laboratories perform experiments that use a variety of substances and materials such as chemicals, nanomaterials, and radioactive substances. Depending on the type of materials and substances, some experiments can pose risks to the facilities, the experimenter, and the environment. Research facilities continuously receive short-term users or experimenters from different countries conducting research experiments. At facilities such as spallation sources and synchrotrons, the environment that the users encounter consists of various experimental stations with associated beamlines and instruments. At an experimental station a user can encounter hazardous contexts that sometimes combine potential exposure to numerous hazards, including radiation, extreme temperatures, chemicals, biohazards, and nanomaterials. Particular focus in such facilities must therefore be placed on safety and the risks associated with users conducting experiments.

Several accidents and incidents have occurred in research laboratories worldwide in the last decade and safety is today a prioritised issue [1]. However, research has also indicated that university laboratories tend to have less developed safety management systems and safety cultures, with lower investment in safety, compared with industrial laboratories and factories [2,3]. Steward et al. [4], comparing safety at industrial and academic laboratories, conclude that there is a higher level of safety rules for work in industrial laboratories. In academic laboratories, the safety rules practised have been found to be more relaxed, and often to depend on the judgement of the investigator in charge [4]. However, academic institutions have also shown a desire to foster positive safety cultures with a commitment to safety, and to integrate safety as an essential element in the daily work of laboratory researchers [5].

A research facility can be viewed as a complex safety-critical system composed of technical, social and human elements. It is a facility where safety needs to be managed adaptively to maintain a safe environment. To increase system safety, a research facility needs an efficient safety management system designed to optimise safe technology, safe human performance, and a safe human-technology interaction and work organisation. An important part of the safety management in research facilities concerns the safety education and training programme for short-term facility users. This safety education should aim at ensuring appropriate knowledge, skills, and safety attitudes among the users. For there to be system safety as well as individual safety, there needs to be a well-designed and efficient safety education programme aimed at the short-term users. However, the goal setting and methods used when developing and ensuring individual-based safety competence can be affected by prerequisites that relate to the socio-technical system in which the education activities take place. Generally, safety-critical facilities must have organisational, technical, and pedagogical prerequisites in order to develop successful learning for safety. Prerequisites can relate to facility design, regulatory demands, the safety culture, pedagogical education, and individual user safety knowledge and motivation.

This paper focuses on the safety management and especially the user safety education of major research facilities such as spallation sources and synchrotrons, the activities of which are based on the generation of radiation for use in research experiments. To our knowledge, the research literature on safety training at facilities using radiation is very scarce, which motivates research on current practices and the identification of risks and suggestions for improvement. Yang et al. [6] conclude that university laboratory safety is still in its early stages of research and belongs to a minority research field when compared with other safety domains.

The study reported in this paper aims to yield more knowledge about the current design and practices of the short safety education and training that users are required to complete to gain access to the facilities. The objectives are to.

• identify preconditions that impact the design of the safety education,
• describe the design and current practice of the short safety education and training of users at the studied research facilities, and
• identify weaknesses and possibilities for improvements.

## 2 Theoretical considerations

The theoretical considerations in the current study are based on a combination of safety science and pedagogy. Three cornerstones in the safety science research area have served as a base in the study. The first cornerstone is the view that the human element or operator is a vital safety barrier and crucial for maintaining safety and resilience [7,8], in this case the user of a research facility. The human contribution involves adaptive problem solving but also human performance in terms of unsafe acts [7]. Well-designed safety education and training for short-term users is a way for a facility to define and to emphasise the proper human contribution to safety in its activities. The second cornerstone focus on the design of facilities with regards to humans, the organisation, and safety. The third cornerstone focus on safety culture, i.e., attitudes and values in an organisation that creates conditions for risk taking and safety performance. The cornerstones are relevant aspects to include from the perspective of designing facility safety education and training. The theoretical considerations in the study are also based on scientific knowledge on good and effective learning pedagogy. For short-term facility users to achieve competence (appropriate safety knowledge, skills, and attitudes), the development of education activities generally requires the completion of a number of steps where the aim is safe performances and safe working environments. Here follows a review of the safety science cornerstones and important parts when developing and implementing education activities.

### 2.1 Facility design

A facility's design phase plays a critical role in enhancing system safety as it sets the requirements for a facility's entire life cycle [9]. In a socio-technical system context where safety training is included, approaches such as safe by design [10], user-centred design [11] and similar principles related to facility design will affect the focus and need for individual-based competence in the system. The principle of safe by design implies striving to make a product or facility as safe as reasonably practicable by working with risk identification and risk elimination from the early design phase [10]. Safe by design should permeate all design decisions – formal and informal – and therefore requires an official and clear mandate to be realised.

Standardising design requirements and following industry conventions reduces the need for site-specific, unique knowledge of personnel and users [12]. This is especially so when there is an exchange of personnel between sites or facilities or, as in this case, when many users visit the research facility to carry out their experiments.

### 2.2 Safety culture

Safety culture has to do with an organisation's ability and willingness to handle risks [13] and relates to the enhancement of the organisational capability to act in a flexible and safety-conscious manner under changing conditions. Hale [14] defines safety culture as “the attitudes, beliefs and perceptions shared by natural groups as defining norms and values, which determine how they act and react in relation to risks and risk control systems”. Wu et al. [15] studied the influence of organisational and individual factors on safety climate in university laboratories and found that both the presence of a safety manager and safety training positively affect the climate. Additionally, Salazar-Escoboza et al. [16] concluded, in their study of safety climate in academic laboratories, that the absence of institutional safety commitments contributes to increased numbers of accidents in laboratories. Hill and Finster [17] highlight the role of leaders at all organisational levels in an academic institution, in establishing and addressing critical elements of a strong safety culture.

### 2.3 Development of education activities

Development of safety education and training generally requires the completion of a number of steps [18]. The safety education and training need to be developed according to the inherent educational needs of the socio-technical system in which humans, technology, and work processes interplay to create results (i.e. the knowledge and skills needed by different users and personnel groups in order to perform various tasks) [18]. The aim should be safe performances and safe working environments. The safety-related competence requirements for individual functions and tasks need to be specified based on the characteristics of the socio-technical system [19]. In order to identify and specify the competence requirements, structured evaluation processes are applied using inputs such as analyses of the facility's design and construction, regulators' requirements and recommendations, risk analyses and risk assessments performed at the facility, event-driven updates (e.g. after facility modification or after an incident or accident has occurred) and unique facility-specific competence requirements (e.g. nomenclature). Once the competence requirements are specified, they are translated into learning outcomes and then designing of the actual safety education and training can begin [18]. In this process, learning activities as well as competence assessment activities are designed in parallel. Finally, the actual safety education and training courses are implemented based on a progression of the individual learners' knowledge and competence.

The safety education should be continuously and systematically evaluated and updated. All the various steps of the process of developing an education activity should be evaluated, as well as the specific contents and the quality of specific education and training courses [19].

### 2.4 Learning outcomes

Based on the competence requirements identified, related learning outcomes are developed. The intended learning outcomes define the goals that will be achieved through the education concerning knowledge, skills, and attitudes, and the qualifications to be gained [20,21]. Four interplaying forms of knowledge are facts, comprehension, skills, and familiarisation [22]. They are relevant both when determining learning outcomes (What kind of knowledge do you want to achieve?) and in designing courses to support learning to achieve them. A course should be defined primarily in terms of its intended learning outcomes [23].

### 2.5 Designing learning activities

Based on the intended learning outcomes of a course, a series of learning activities are designed and organised to support users or course participants in developing knowledge, skills and attitudes that meet the intended learning outcomes [24].

For the learner to experience meaning and context, it is important that the learning takes place in stages and starts with an overall perspective where the end goal is in sight, and then practising on different parts that make sense in this larger perspective [25]. The learning activities should allow enough time to result in long-term learning. Learning that is spread out over a longer period of time, with spaced practice and with some variation in the arranged learning situations [26], gives the learner a better chance to build adequate memory traces. Well-designed learning activities have focus and variation. In a particular learning situation, arranged or spontaneous, what the learner's attention is directed towards is crucial. We learn best what we pay attention to and become aware of, because this focuses our mental energy and usually our conceptual thinking. This supports the organisation of our memory, which in turn facilitates recollection. According to the variation theory of learning [25], intelligent action is based on the individual being able to discern relevant aspects in a given situation in the form of dimensions of possible variation [27]. Which of these dimensions a particular individual can discern depends on the individual's past experience [25].

Learning activities should also provide motivation (associated with attitudes and values) and encourage intentional learning. Marton and Booth [25] state that successful educational processes require the individual to experience the meaningfulness of the education at large, and of each activity of the education in particular. The individual's motivation for learning affects their willingness and ability to take on learning activities and increase learning.

### 2.6 Examination design

After completing a course, an examination is a way of determining the extent to which an individual has achieved the defined learning outcomes and competence requirements [28]. Determining whether individuals have the required knowledge and skills necessitates valid examination methods designed according to the type of intended learning outcomes. The examination design is important as individuals tend to adapt their learning efforts to the examination that will take place [29]. Examination assignments can be knowledge-testing or knowledge-developing [23]. Depending on the purpose of the examination, the questions can be about facts, understanding, or knowledge development. Examples of knowledge-developing questions are: “Is there a built-in safer way to do this?” and “What more could you do to avoid risks at this stage of execution?”

Summative examinations are a type of exam that examines retrospectively at the end of a course or educational programme. By contrast, formative examinations are taken on an ongoing basis during the learner's education. The examination tasks have a prospective purpose, which is to support the learning through feedback on the results achieved so far [30]. Formative feedback is very valuable in education, not least in the safety context, as it facilitates individual improvements and further development.

The pedagogical literature provides many specific tips on the direct formulation of examination questions and choice of question types. Generally, important aspects to consider in formulating examination questions are: formulate the question in plain terms; formulate the question so that the individual needs to think and formulate the answer themselves; avoid formulating yes-or-no questions (they require little of the individual and there is a high chance of a correct answer); and vary the way the questions are asked.

### 2.7 Evaluation of safety education activities

The safety education and training in any safety-critical organisation needs to be continuously evaluated and updated to correspond to the adaptive development of the organisation and to reflect the total socio-technical system. The continuous evaluation and updating of processes are fundamental in a learning organisation [31]. Learning concerns the improvement of practices and routines [32] and is a key process for improving safety in organisations [33]. The continuous evaluation and development of safety education activities requires systematic and well-thought-through processes. The evaluation consists of several layers, from evaluating the entire education activity, to evaluating the specific contents of safety courses, and evaluating the quality of courses and checking that course objectives are met [19].

The evaluation of the entire education activity should be based on data collected in all the steps of the process of developing the activity. This provides the opportunity for feedback that can lead to improvements in all parts of the process of developing an educational system. The continuous evaluation and development also includes the contents of specific safety education and training courses taking the socio-technical system safety perspective into account.

## 3 Materials and methods

To gain knowledge about the current design and practice, identify preconditions as well as weaknesses that impact the safety education and training of users, an exploratory stance in the data collection methodology was taken. The exploratory stance is justified by the early stage of the research field and few existing studies. The methodology consisted of site visits to three research facilities using radiation, semi-structured interviews with individuals having specific functions at the facilities, and review of facility documentation. This was then analysed using content analysis (thematic analysis and category coding).

### 3.1 The three studied facilities

The three studied facilities were Synchrotron I, Synchrotron II and Spallation I. Synchrotron I is a Swedish national research laboratory situated at a Swedish university. The facility produces synchrotron X-rays of very high intensity and quality. The short wavelengths of synchrotron light enable visualisation of details that are otherwise impossible to see in materials. The number of users performing experiments at the site will be close to 2000 a year by 2026. At the time of this study, the new Synchrotron I facility had been built but was not yet operational. Furthermore, the old facility was still in use.

Synchrotron II in France is a synchrotron radiation research facility with the world's most intense X-ray source. The facility is an international cooperation between 22 partner countries. Following 30 years of operation, it has a new standard for synchrotron storage rings – high-energy, fourth-generation storage rings – with unique X-ray performance. Each year, about 9000 scientists from around the world come to the facility to conduct experiments at the beamlines.

Spallation I is a European research facility currently being built in Sweden. It is based on one of the world's most powerful neutron sources. The neutrons will be used to analyse different types of materials at the atomic or molecular levels. It is estimated that the facility will be ready for operation in late 2027 and when in full operation an estimated 2000 to 3000 researchers annually will conduct experiments at the facility. Thus, the facility was not in use when this study was performed.

### 3.2 Data collection

#### 3.2.1 The site visits

The site visits at the three research facilities gave rich contextual and background data about the activities and organisation of the facilities, working and safety procedures, and safety education and training of users and experimenters. The information and data from the visits functioned as a point of reference or back drop for the qualitative analysis of interview data.

At Synchrotron I, site visits were conducted both at the old facility, which was still in operation at the time, and the new Synchrotron I facility, which was not operational but built. By being able to visit both the old and the new sites, a very clear description of how, based on experiences from the old site, the safety work with the new was being developed. It also gave valuable information on the design and use of the safety education. In addition to the site visits, the facilities were visited several times to conduct the semi-structured interviews.

The field study at the Synchrotron II facility in France was a full 2-day visit including both a tour of the facility and interviews. The visit provided valuable knowledge about how experiments are carried out in these types of facilities, as well as on the design and use of the safety education of users.

As Spallation I had not yet been completed at the time of this study, the site visits consisted of interviews with relevant actors who worked with various aspects during the construction of the facility, primarily with a focus on safety.

#### 3.2.2 Facility documentation

The site visits were supplemented with reviews of documentation received on the facilities' safety work and education and training, as well as with information on the facilities’ home pages. The documentation gave information on the user process of applying for experimental time at the facility, the introductory safety training of users, radiation protection courses, and radiation protection courses specifically in connection to beamlines/instruments. The information on courses also contained the subject areas included. The documentation gave information on the safety organisation and accident prevention, and on staff functions at the facility. The document review gave background information that enriched the understanding, analysis, and interpretation of collected interview data.

#### 3.2.3 Interviews

A total of 20 exploratory semi-structured interviews were conducted at the three studied research facilities. At the two Swedish facilities, interviewees were approached through the facilities’ respective chief secretary, who was informed about the aim of the current research project and the request to reach individuals at the facility for interviews. The secretaries identified individuals and got their consent to participate in the research project and scheduled the interviews. The French facility was contacted directly by the research group. Table 1 presents the number of individuals interviewed at each organisation, and their functions. The number of interviewees is considered sufficient as they all had great experience in designing and using these types of research facilities. The interviewees had various functions related to the design, development and operation of the facilities. Not all interviewees were directly involved in safety education, but they provided expert information as a basis for the training. Most had own experience of short safety training when visiting similar facilities around the world.

**Table 1:** The 20 individuals interviewed at the three studied organisations, and their functions.

Synchrotron I	Synchrotron II	Spallation I
Director Machine Director Head of Safety & Work Environment Head of Linear Accelerator Manager of User Office Radiation Safety Expert Gas Manager Safety and Work Environment Officer	Head of Safety Group Classical Safety Engineer Radiation Protection Engineer	Director General Director, Environment, Safety, Health & Quality Head of Environment, Safety & Health Division Head of Quality Division Head of Accelerator Division Head of Neutron Instrument Division Radiation Safety Expert Senior Security Officer Safety Training Officer

All interviews were conducted on site in the facilities and all but two interviews were recorded. The reason these two interviews were not recorded was that the informants did not want it. All interviews but especially the two unrecorded interviews were documented with notes. During most interviews all three researchers participated. For each interview one of the researchers were responsible for leading the interview, while the others had the role of asking follow-up questions that the primary interviewer may have missed. Interviews took 1–1.5 h.

The interview protocol was related to the research objectives and consisted of two main areas. The first area covered questions on what the interviewee worked with at the studied research facility, as well as on earlier experiences of other research facilities. If the interviewees described having experiences with other facilities (which most of the respondents did), they were also asked about experiences with the safety education and training, the processes of conducting experiments, and/or working at this or these facilities. The second area focused on safety and safety education, both on its current practice and how the interviewees understand future needs. The semi-structured interviews covered predefined themes and questions in the interview protocol, at the same time being open to changes and follow-up questions. The exploratory stance gave more openness and allowed for new issues or complex problems to be introduced in the interviews to better describe or define them [34].

### 3.3 Analysis

Interview data, notes from study visits and interviews, as well as the collected documents were analysed using thematic analysis and category coding [[34], [35], [36]]. The analysis related to the research objectives and was initially based on predefined themes from theoretical pedagogical and safety frameworks, as well as on practices of education design and implementation. More specific, the following themes were included: the (radiation) safety concept, safety culture, proactive safety work, learning for safety, safety education systems, experiences and challenges concerning safety education and training, pedagogics, subject didactics, examination methods, processes for quality and development, and networks and collaborations.

The data analysis had an inductive approach where all three researchers conducted the analysis in a flexible and highly interactive process both with the data and between researchers. Several analysis steps were conducted to identify categories and themes from the data. Each researcher carefully listened through the recorded interviews and read the interview notes and made descriptive notations. At the beginning, the same five interviews were analysed by each researcher and comparison of notations were thereafter made. The notations were coded into preliminary categories. The coding was an iterative process were the researchers had a continuous dialogue about the data and notes, and during regular meetings the researchers' codings were checked for inconsistencies. The remaining fifteen interviews were analysed and coded using the categories, but categories were also added and refined along the way, and sub-categories were in some cases developed. Each researcher also read through the documentation and notes from the facility site visits. The information was coded using the categories, and similarities as well as differences in information compared with interview findings were considered. However, very few differences were found. Comparisons and small adjustments between researchers’ category codings were thereafter performed and resulting themes were formulated.

#### 3.3.1 Ensuring quality of research and findings

In general, when assessing the quality of research, objectivity, reliability, and validity are important research quality criteria. Objectivity has to do with findings being the same regardless of who performs the research or data analysis. Reliability has to do with your data or findings being free of error. Validity has to do with the extent to which you succeed in ‘measuring’ what you are set out to measure, i.e., that the category coding captures what you are set out to capture [35]. Concerning reliability, the opinion of qualitative researchers seems to differ were some reject it, accept it, or suggest a modified concept of reliability [35]. In the modified concept, the systematic way of performing research and showing how you arrived at your conclusions is highlighted [35]. In the current case, the researchers had a systematic approach when performing interviews at the three sites, and specifically when analyzing the data material.

Schreier [35], states that objectivity and reliability play a larger role in qualitative content analysis than it does in qualitative research in general. Objectivity of the category coding can be checked by having another researcher interpret and code the data material. This double-coding also assesses the quality of the coding, e.g. if categories are clear and not overlapping, and therefore if it is reliable. In the current research and analysis, the three researchers performed the category coding in parallel in a systematic and iterative process and arrived at very similar interpretations of the data, which accounts for objectivity and reliability in the analysis.

In qualitative research, validity is crucial as the research is conducted in natural settings, and the research is data-driven which means conclusions are based on interactive and close reading of the data material [35]. Validity of the data interpretation and category coding is achieved by the adaptation of categories to the data, and of the extent that the coding categories represent the focus of the research questions. When the data analysis has an inductive approach, as in the current case, face validity is important when wanting to validate if a category coding describes the data material [35]. Indications of low face validity is when the category coding does not cover the meaning of the material and there is a residual category describing large parts of the data material, and if there are high coding frequencies for one sub-category compared to other sub-categories [35]. When analyzing the current data material, the researchers were able to formulate and refine categories and sub-categories that describe the data and very little information ended up in a residual category. The researchers also believe that the current level of abstraction of the categories and themes is appropriate leading to informative and understandable categories and themes and that they also fit together.

No major differences in resulting themes and category codings were expected for the three (Swedish and French) studied research facilities. This was also the outcome, and we believe that the research has been able to capture a general existing order in these types of facilities regarding the safety education and training and related issues. The site visits showed that there was an exchange of both material and personnel between these types of facilities around the world and it was described as common to exchange e.g., safety course material between facilities.

Based on the analysis, aspects and themes were identified that either have influenced or should influence the development and implementation of the education design. These aspects are presented in the results section.

## 4 Results

Following the objectives of the study, results will be presented focusing on a) preconditions which the facilities had very little influence on but which impacted the design of the safety education; b) aspects in the design and current practice of the short safety education and training of users at the facilities; and c) apparent weaknesses that emerged in the interviews or documents, weaknesses that are not sufficiently taken into account in the design of current training courses. Table 2 presents an overview of the results.

**Table 2:** Overview of results on preconditions affecting education design, aspects in current design and safety education practice, and found apparent weaknesses.

Preconditions affecting education design	
Regulator demands	Facility licence required and compliance to regulations
	Requirement to apply a systematic development approach to safety education and training
Heterogenous user groups	Pedagogical challenge when safety and safety culture background knowledge vary
	Search for new user groups from industry and academia will require greater user support
	Required knowledge, skills, and attitudes must be ensured
	Avoid repetitious education as it severely affects learning motivation
	Determine on individual level which learning outcomes for achieving radiation safety need to be tested and at what intervals

Aspects in current design and practice	

Similar format of safety education and training across facilities	Generally: - on-line general safety course; -e-education on specific beamline/instrument; and - practical introductory training led by personnel at instrument.
A necessary evil	Short safety education often perceived as neither motivating nor meaningful
	Integration of safety course with regular education and training may render increased internal motivation
Facility design	Safe-by-design, user-centred design, affect the focus on individual-based safety competence, however unclear how the approaches controlled facility design
	A basic idea found: it should be difficult to make errors and mistakes
	Philosophies allowing high/low user interaction with facility systems affect the amount of user safety education
User's safety background knowledge and competence requirements	Must be identified e.g., through submitted application for experimental time
	Assessment of user's need for safety education could be improved
Facility safety culture	Major safety culture challenges with short-term users of many nationalities
	Safety culture aspects must be included in safety education, however unclear how knowledge about aspects were communicated
	Use of facility role models was successful, e.g., the personnel receiving users at beamlines/instruments
	Safety culture-enhancing activities are important
Continuous improvement and development of safety education	Analysis processes for risk and safety also contributes to development of safety education activities
	Feedback from users, contractors, trainers, and facility personnel important
	Networking and collaboration between facilities exists, however, no formalised safety knowledge exchange

Apparent weaknesses	

User learning outcomes	Processes for identifying and developing learning outcomes could be improved and deepened
	Learning outcomes on factual knowledge, safety rules, and compliance, but lacking on safety principles
	Examinations were mainly factual knowledge-testing, not knowledge-developing
Pedagogical education of educators	Facilities' radiation safety experts produced the e-learning and examination
	Knowledge in pedagogy, didactics, and educational planning was lacking
	Generally, a low pedagogical awareness in the sector, which limits the effectiveness of safety education activities

### 4.1 Preconditions affecting education design

#### 4.1.1 Regulator demands

One precondition or prerequisite concerns regulator demands. The studied facilities all need permission (a licence) from a (radiation safety) regulator to conduct their operations. As licensees for.

Activities involving radiation, they are required to comply with regulations on optimising radiation protection and safety, limiting exposures, and implementing radiation protection technologies in the workplace [37,38]. Regulators can also require that safety education and training courses are systematically developed (e.g. using the systematic approach to training [SAT]) [19]. The SAT enables traceability, that is, the ability to show that, on the basis of an educational need, and as a result of the subsequent design and implementation of an education or training course, the individuals will have obtained the specific knowledge required to meet the corresponding need [19]. In the current case, the two research facilities in Sweden (and also the French facility) were required by the regulator, the Swedish Radiation Safety Authority (the French Nuclear Safety Authority), to apply a systematic approach when designing and maintaining safety education and training programmes.

#### 4.1.2 Individual user safety knowledge and learning outcomes

Another precondition concerned the heterogeneous user group. The users of the research facilities have different nationalities, different background knowledge and training, and heterogeneous prior knowledge about safety and safety culture aspects. Some of the users are experienced researchers who have visited other, similar research facilities and have undergone basic facility-specific education in, for example, fire, chemical and radiation safety in various places. Other users are inexperienced and first-time visiting researchers. In addition, our interviews showed that the facilities were actively working on identifying new user groups from both industry and research, that is, users who had no prior experience of working in these kinds of environments and therefore would require greater effort on the part of the facility, for example in the form of extra support. Therefore, individual safety expertise varies widely.

Designing safety education that meets the need of users with varied safety background knowledge was described by the interviewees as a pedagogical challenge. For new users, it is essential that the training course includes basic safety knowledge. However, an important consideration when it comes to experienced users is to not include extensive repetition of basic knowledge, but to carefully determine which learning outcomes need to be tested and at what intervals. One interviewee emphasised the importance of avoiding repetitious education as this severely affects the individual's motivation. As described by the interviewees, having an education course that will suit everyone is a challenge.

### 4.2 Aspects in the current design and practice

#### 4.2.1 User education and training similar across facilities

The format of the short safety education and training for users was described as similar across facilities around the world. Generally, and as a standard at international research facilities with radiation sources, the first part of the education activities for facility users consists of a short e-based safety education that must be completed before access to the facility is granted. These courses are focused primarily on radiation safety, but also touch on other safety areas such as fire, chemical, and electrical safety. As the interviews indicated, the e-education often consisted of a PowerPoint presentation (ranging from 30 min to 2 h) and a multiple-choice test. Taking Synchrotron I as an example, after the general introductory safety course online, the training continues with an e-education containing information on the specific beamline or instrument where the user is to perform an experiment (as all beamlines and instruments are different). In addition to the e-learning, users are usually given a practical session at a specific experimental station in order to gain access and carry out their experiments.

#### 4.2.2 A necessary evil

An aspect that emerged was that the current short education is not motivating. Interviewees indicated that the general perceptions among users about the e-based safety education are that it is not pedagogically based, which diminishes the sense of meaningfulness, rendering it pedagogically inefficient with regard to learning. Related to this, the interviewees' prevailing view of users’ attitudes to obligatory safety education was that users understood the intention behind it, yet often saw it as a necessary evil, to be completed quickly and without much effort. One interviewee suggested that a way to increase internal motivation was to integrate the safety course with the “regular” education and training that short-term facility users get on the use and handling of instruments at the facility.

#### 4.2.3 Facility design and need for individual-based competence

As mentioned, approaches such as safe by design, user-centred design, and similar approaches related to facility design will affect the focus on and need for individual-based competence in the facility. In practice, it is a way to reduce the amount of content to be included in the education and training. Several interviewees at the research facilities expressed ideas in line with the concept of safe by design, but it was unclear to what extent this approach really controlled the design and construction of the facilities studied. However, one basic idea when designing Synchrotron I was that it should be difficult for an individual to make errors and mistakes; for example, they used colours for certain objects that should not be interfered with to maintain the radiation protection.

In relation to safe facility design, some interviewees gave an international perspective on accelerator facilities and highlighted two existing philosophies regarding the facility's management of users. One philosophy gives more room for users to interact with the facility's systems. The other philosophy gives much stricter conditions for the users' interaction with the technology, where some actions are handled solely by the permanent local staff or by automated systems. This reduces the amount of user education and training and also makes it easier when users have different background knowledge. For example, comparing the new Synchrotron I facility to their old facility, the ability for users to rebuild and adapt the experimental stations has been reduced. Likewise, the latter philosophy was applied in the design of Spallation I.

#### 4.2.4 User's safety background knowledge and competence requirements

An important aspect emerging from the interviews is the identification of users' safety background knowledge. A user's safety background knowledge and competence requirements could in part be identified based on the safety review of the proposed research experiment the user planned to perform at the facility. Before a user can conduct an experiment at a research facility, an application for beamtime (experimental time) is submitted to and assessed by the facility. The process for applying for beamtime was described as similar across facilities. Applications are reviewed according to three factors related to the proposed experiment: feasibility, scientific value, and safety. In order to obtain beamtime, a proposed experiment has to be approved on all three. If specific risks are identified, an additional detailed risk analysis and evaluation is performed using an iterative process including the user and the facility safety group. Nevertheless, interview results revealed that the assessment of individual applicants' needs for education and training in safety could be further improved.

#### 4.2.5 Facility safety culture and short-term users

In the current case, the facility safety culture was described by several interviewees as a major challenge with regard to safety and safety work at a facility. As previously mentioned, the personnel and visiting users at such a facility are of many nationalities and reflect the safety and work cultures they come from. One interviewee emphasised that safety culture aspects must be included in the safety education provided at the facility, but it was unclear how knowledge of these aspects was communicated at the facilities. However, the use of role models was seen as a successful way of showing how to behave correctly, not only when it came to technical aspects of safety, but also with regard to norms and attitudes. An example of important role models was the personnel who received the users at the beamlines/instruments. In the interviews, they were highlighted as especially important for safety and the safety culture at the facilities. The practical display at beamlines and experimental stations serves as safety training (through developing relevant skills) and practical operations training, and as a safety culture-enhancing activity (if compliance with rules is emphasised and safety considerations are clarified). The results also showed that the Synchrotron II safety group conducted a daily general safety course at the facility, aimed at contractors and short-term visiting groups. This meant that all contractors met a representative of the safety group. This was described as having a positive effect on safety motivation, communication, and safety participation.

#### 4.2.6 Continuous improvement and development of safety education

Various analysis processes in an organisation contribute to the evaluation and development of the organisation's safety and its safety management and also contribute to the development of its safety training activities. Examples of such analysis processes mentioned in the interviews are risk analyses, incident and accident reporting, safety rounds, and safety audits. Analyses that contribute to the development of safety education must be based on experiences gained in practice by users and contractors and through direct feedback from facility personnel such as trainers of safety courses and those responsible for safety briefings at a beamline or instrument. Interviewees at Synchrotron I described that when users had carried out an experiment, they completed a feedback form giving information, for example, on how the experiment had been carried out and on whether they had felt safe during the execution; as well as on practical things such as whether they had found the guest house clean. The feedback also included comments relating directly to the education and training.

There were similarities between how the three facilities worked with safety issues. Collaboration between the various facilities existed. An international network has been created where research facilities with radiation sources exchange knowledge through advisory committees on, for example, safety, health, and environmental issues. However, no formalised knowledge exchange exists, in contrast to the nuclear power industry, where adverse events are reported through formalised systems to central units promoting learning for safety.

### 4.3 Apparent weaknesses

#### 4.3.1 User learning outcomes

One apparent weakness concerned the learning outcomes. Interview results showed that the facilities had processes for identifying and developing learning outcomes but that there was room for designing more nuanced and thought-through outcomes. The learning outcomes focused on factual knowledge, safety rules, and compliance. Learning outcomes regarding knowledge of safety principles such as the ability to carry out risk assessments were, however, largely lacking.

At the facilities, the examination assignments were mainly knowledge-testing (and not knowledge-developing) – in other words, factual. As the interviews indicated, the e-education often consisted of a PowerPoint presentation and a multiple-choice test. If you failed the test, you could take it again. Passed examinations, which are facility-specific, are generally valid for 1 year, after which, the individual is required to renew the course (or at least retake the test).

#### 4.3.2 Pedagogical education of educators

A second apparent weakness concerned the pedagogical training of the educators. The study results show that the facilities' radiation safety experts produced the e-learning units with PowerPoint slides and examination questions that were used in the research facilities' safety education and training. Experts in other fields at the facilities also provided expert information as input and as a basis for the education and training programme. However, in order to organise and implement education activities, adequate expertise in pedagogy, didactics, and educational planning is also required but was lacking.

In the study, it was often unclear whether, and to what extent, the facilities clearly distinguished between intended knowledge forms, intended learning outcomes, the actual content of a course, and achieved learning outcomes. One interviewee, a member of a safety group, said that they had access to an “instructional designer” who developed and designed teaching materials for e-learning. However, in general, and as the interviews revealed, the current practice in the field reflects a low degree of pedagogical awareness, which limits the effectiveness of safety education activities.

## 5 Discussion

Research facilities such as spallation sources and synchrotrons have existed for several decades, but the research on the short safety education and training required for users to gain facility access is limited. This study aimed to yield more knowledge about current preconditions that impact the safety education design, about the design and current practice of the safety education, and about weaknesses and possibilities for improvement. The education consisted of e-learning and a practical introduction at a beamline or instrument.

Safety education and training is one way of promoting system safety in complex facility environments, and to ensure appropriate safety knowledge, skills and attitudes among facility users. Adoption of approaches such as safe by design and user-centred design in a facility's design stage plays a critical role in meeting the need for individual-based safety education and training.

Two of the research facilities studied were newly built and a tendency to have an often one-sided focus on the newest technology in order to produce the best research results was described. All too often, the importance of also focusing on the people and organisational issues around the technology was forgotten. Safety-critical facilities must have the necessary competence and skills to understand and manage the combination of people, technology, and organisational factors in order to reduce the risks of designing less well-adapted facility solutions.

Perceptions on safety education, among users, were that it was a necessary evil to be completed quickly and without too much effort. This finding is in line with Czornyj et al. [39], who report that “researchers are generally more focused on scientific progress in their field […] than [on] learning new topics in laboratory safety”. Users’ external motivation to gain facility access, rather than interest in increasing risk awareness, may signify a surface-oriented approach to learning based on a desire to pass a test rather than a willingness to learn [41]. External motivation is unfavourable for much of the learning that should take place during safety education. One way to increase internal motivation among users is to integrate safety education with the “regular” training on the use and handling of instruments, which takes place before the users are allowed to use the experiment station at the facility. A strong advantage of this approach is that users are usually highly motivated to learn how to perform their experiments, while safety rules and routines can be perceived as inhibitory and are therefore less motivating to learn. A new view that safety training is needed and useful needs to be created in the community.

### 5.1 Safety culture

The research facilities under review have a continuous flow of short-term users and visitors, which can lead to instability in a facility's safety culture. Actively upholding a good safety culture is essential for safety and the large number of visitors highlights the need for facilities to perform continuous safety culture-enhancing activities, and to communicate to users the characteristics of the safety culture they (the facilities) strive for. The e-learning for users needs to include safety culture aspects that are presented pedagogically. Clear safety expectations must be established for users, and also for facility personnel. The facilities need to have processes for accomplishing this. They need to have people working actively with safety culture-promoting activities. The safety education and training of personnel in these matters is also crucial.

The practical introduction at a beamline or instrument was considered by the interviewees as an especially important part of the safety education and of maintaining a safety culture. The advantages of integrating safety into these introductions are, for example, the ease of learning practical elements, an increase in the participants’ motivation, and a strengthening of competence development.

### 5.2 Learning activities

Variety in learning situations increases the effectiveness of absorbing abstract written material [19]. Learning activities taking place in the actual facility can have stronger learning effects compared with abstract communication. In situ activities may better capture the course participants' attention and provide stronger cognitive memory traces (recall) than an abstract communication based on speech or text and focused on conceptual aspects. Learning activities must have a clear context and purpose, which means there needs to be an understandable context for what is to be learnt, in order for meaningful knowledge to develop in the individual. Here, a distinction can be made between physical and social contexts [41,42]. The physical context of the learning activity mimics the context for which it is to prepare. The social context is about the individual's interaction with others, for example by building common knowledge through collaboration and dialogue. The requirements for appropriate safety attitudes are to create a consistent safety culture within a facility. Safety culture aspects can be included in a facility's safety training in various ways. These include theoretical reviews with examples of practical application, written reflections, and meetings directly with operators (role models) in the facility. The use of role models is an effective way to increase safety and a successful educational tool in safety education and training. The role models, such as beamline managers with safety responsibility who supervise users at a beamline, have a direct impact and positive effect on users' safety motivation, safety behaviour, communication, and execution of experimental tasks.

In the current case, learning activities can be advantageously spread out, with learning elements before (e.g. e-learning), in connection with, and during the stay at a facility (the learning perspective before, during, and after an actual course). Based on a learning process, the “before phase” can be described as theoretical preparation (e.g. reading materials, writing reflections, viewing instructional films). In the “during phase”, activities take place that can be teacher-led and start with a reflection on what was learnt in the before phase. In the “after phase”, some form of post-production occurs, such as practical experimental execution. Stepwise learning activities are preferred in order for the learner to experience meaning and context [25].

### 5.3 Assessment of individuals’ educational needs

The interviews in this study showed that the assessment of individuals' needs for education and training in safety could be further improved. One way would be by initiating an individual user's journey through a facility's safety educational system with diagnostic tests. In this way, the organisation could individually adapt the education and the individual users themselves can take control of their learning based on clear needs and requirements. To be able to determine individuals' level of safety-relevant competence, some form of central documentation can be made available on the training and examinations the individuals have completed, and on how long these are valid for. When revising a facility's competence requirements and learning outcomes for courses, individuals in need of supplementary training can also be identified.

For new users, it is essential that the education and training include basic safety knowledge. However, an important consideration when it comes to experienced users is to not require extensive repetition of basic knowledge, but to carefully determine which learning outcomes need to be tested, and at what intervals. It was found that motivational challenges arose among users who were faced with compulsory safety education, but already met the required levels of ability in safety training they had undergone previously. Alternating between different pedagogical methods will better maintain the individuals’ motivation and increase opportunities for learning.

### 5.4 Knowledge-developing examination

At the three facilities, the examination assignments were mainly of a factual knowledge-testing kind. An analysis showed that the examination methods could be further improved in parallel with the development of the learning outcomes. The examination could be designed to be more interesting and thus to attract a quest for knowledge. Above all, it could be designed to present an opportunity for the individual both to better structure their knowledge and skills, and for reflection. The International Atomic Energy Agency (IAEA) [43] and Czornyj et al. [39] emphasise increased knowledge of safety principles in safety education, which concerns the abilities to carry out risk assessments and propose risk mitigation measures. They believe that educational and training materials for laboratory safety often lack specific examples or real-life scenarios. A learning outcome in a laboratory safety course may be to identify risk sources associated with specific protocols and experimental procedures, which develops skills in risk assessment [40].

### 5.5 Continuous improvements

The safety education and training in the facilities needs to be continuously evaluated and updated to correspond to the adaptive development of the socio-technical system. The evaluation should include an assessment of individuals' competence needs in relation to the facility's safety status. Various types of analyses contribute to the development of safety, safety work and concurrently the organisation's safety education activities. Important processes are risk analyses at the facility, feedback on incident and accident reporting, both in one's own and in other facilities, and information received from safety rounds and safety audits. Feedback is also important on users' practical experiences when conducting experiments, contractors' experiences when performing work and, not least, information from trainers or instructors who conduct safety courses and training. Evaluations and audits should immediately affect the content of the specific courses and education activities, so that they are adapted to changing circumstances and new insights.

### 5.6 Pedagogical resources

Safety-critical facilities with education activities must have sufficient resources to develop, implement and maintain learning activities for users. To do this, they also need to provide pedagogical continuing education for safety educators at the facilities. A facility's experts (such as radiation safety personnel) have important roles in setting safety competence requirements and learning outcomes, and in recommending course content, which also creates a need for pedagogical expertise that may not exist within the organisation. Yang et al. [6] highlights the advancement and promotion of worldwide university laboratory safety through international cooperation among research institutions to share knowledge and experiences, good practices, and lessons learnt.

### 5.7 Implications for research and practice

Research in the field of safety education at research facilities using radiation is very limited and this study has contributed with knowledge on current practice. The study points to knowledge gaps and further need for research on the pedagogical development of short safety education, on the identification of individual knowledge needs, and on how safety culture aspects best can be communicated. The study has identified possibilities for improvement which is believed to be of worth to practice and also when taking an international research facility perspective. The study results can support the design of safety education and training in these types of research facilities as well as the development of system safety.

### 5.8 Limitations of the study

This study is based on just three research facilities in two countries. However, most of the interviewees had either worked or performed experiments at other facilities (spallation sources and synchrotrons). Thus, most of the interviewees had first-hand experience of short safety training when visiting similar facilities around the world. Which means interview data deals with more than just the facilities studied.

From an international perspective, employees at this type of facilities seems to work very closely together with employees at other similar facilities. For example, when it comes to safety aspects, expertise is described to be shared to support each other in designing safe facilities and also when developing education materials. There are also international workshops where employees from these types of facilities meet regularly and discuss safety issues.

The study design did not involve actual users at the facilities and the study would have been strengthened if we deliberately interviewed users. Still, most of the interviewees had experience of being users at other facilities.

## 6 Conclusions

Safety education and training is important for safety and resilience in complex facility environments such as spallation sources and synchrotrons, and to ensure appropriate safety knowledge, skills, and attitudes among facility users. The objectives of the study were to identify preconditions that impact the safety education design, to describe design and current practice of the safety education of users, and to identify weaknesses and possibilities for improvement. Study objectives were adressed in 20 exploratory semi-structured interviews with individuals with relevant functions at two Swedish and one French research facility, as well as in reviews of facility documentation.

Study conclusions were.

• Sufficient organisational resources and competences are needed to develop and maintain educational activities, and to provide continuing pedagogical education for facility educators.
• Continuous safety culture-enhancing activities need to be maintained to meet the challenges of having large numbers of short-term international users at these facilities.
• Safety values and behaviours can be enhanced through users' interactions with personnel and through role models at the facilities' experimental stations. Integrating safety education with regular education on the use and handling of instruments at an experimental station is another way to increase users' internal motivation to learn.
• When designing safety education, increased focus should be on the actual competence needs and the mapping of these needs in relation to individual users' competence and their need for more competence.
• Examination methods can be improved to be more interesting and reflective, and can be developed in parallel with the learning outcomes.
• Continuously evaluating and updating the safety education and training activities will promote the development of adaptive and resilient facilities where users with updated safety knowledge form an important safety barrier.
• Research facilities with radiation sources would benefit from having a structured exchange of information on the design of education activities, although some aspects will need to be adapted to each specific facility and organisation.
• There may be a need to move away from the conventional ways of designing and providing safety education and training, and to improve certain aspects through new thinking and innovation. The design and development will also benefit from taking on a socio-technical system perspective as well as a system safety perspective.

The study is limited by only being based on data from three research facilities. However, most of the interviewees had first-hand experience of short safety education when working at or visiting similar facilities around the world. Study design would have been strengthened by also interviewing actual users at the facilities, however, most of the interviewees had experience of being users at other very similar facilities.

## Funding

This work was financially supported by the 10.13039/501100011759Swedish Radiation Safety Authority [grant number SSM2017-2703]. The funder took no part in the design of the study or the collection, analysis or interpretation of the data.

## Ethics declarations

Review and/or approval by an ethics committee was not needed for this study because the data collected and handled did not concern personal data or other kinds of sensitive data. All participants provided informed consent to participate in the study. All participants provided informed consent for the publication of anonymised interview results.

## Data availability statement

Data associated with the study has not been deposited into any publicly available repository. Data will be made available on request.

## CRediT authorship contribution statement

**Åsa Ek:** Writing – review & editing, Writing – original draft, Project administration, Methodology, Investigation, Funding acquisition, Formal analysis, Conceptualization. **Kerstin Eriksson:** Writing – review & editing, Writing – original draft, Methodology, Investigation, Funding acquisition, Formal analysis, Conceptualization. **Jonas Borell:** Writing – original draft, Methodology, Investigation, Funding acquisition, Formal analysis, Conceptualization.

## Declaration of competing interest

The authors declare that they have no known competing financial interests or personal relationships that could have appeared to influence the work reported in this paper.
